# Identification of Key Biomarkers and Immune Infiltration in Systemic Juvenile Idiopathic Arthritis by Integrated Bioinformatic Analysis

**DOI:** 10.3389/fmolb.2021.681526

**Published:** 2021-07-14

**Authors:** Min Zhang, Rongxin Dai, Qin Zhao, Lina Zhou, Yunfei An, Xuemei Tang, Xiaodong Zhao

**Affiliations:** ^1^ Department of Pediatric Research Institute, Ministry of Education Key Laboratory of Child Development and Disorders, National Clinical Research Center for Child Health and Disorders, China International Science and Technology Cooperation Base of Child Development and Critical Disorders, Children’s Hospital of Chongqing Medical University, Chongqing, China; ^2^ Chongqing Key Laboratory of Child Infection and Immunity, Children’s Hospital of Chongqing Medical University, Chongqing, China; ^3^ Department of Rheumatology and Immunology, Children’s Hospital of Chongqing Medical University, Chongqing, China

**Keywords:** systemic juvenile idiopathic arthritis, diagnostic biomarker, mitogen-activated protein kinase, platelet, treatment

## Abstract

Systemic juvenile idiopathic arthritis (sJIA) is a rare and serious type of JIA characterized by an unknown etiology and atypical manifestations in the early stage, and early diagnosis and effective treatment are needed. We aimed to identify diagnostic biomarkers, immune cells and pathways involved in sJIA pathogenesis as well as potential treatment targets. The GSE17590, GSE80060, and GSE112057 gene expression profiles from the Gene Expression Omnibus (GEO) database were screened to obtain differentially expressed genes (DEGs) between sJIA and healthy controls. Common DEGs were subjected to pathway enrichment analysis; a protein-protein interaction network was constructed, and hub genes were identified. In addition, functional annotation of hub genes was performed with GenCLiP2. Immune infiltration analysis was then conducted with xCell, and correlation analysis between immune cells and the enriched pathways identified from gene set variation analysis was performed. The Connectivity Map database was used to identify candidate molecules for treating sJIA patients. Finally, quantitative reverse transcription-polymerase chain reaction (qRT-PCR) was carried out, and the GEO dataset GSE8361 was applied for validation of hub gene expression levels in blood samples from healthy individuals with sJIA. A total of 73 common DEGs were identified, and analysis indicated enrichment of neutrophil and platelet functions and the MAPK pathway in sJIA. Six hub genes were identified, of which three had high diagnostic sensitivity and specificity; ARG1 and PGLYRP1 were validated by qRT-PCR and microarray data of the GSE8361 dataset. We found that increased megakaryocytes and decreased Th1 cells correlated positively and negatively with the MAPK pathway, respectively. Furthermore, MEK inhibitors and some kinase inhibitors of the MAPK family were identified as candidate agents for sJIA treatment. Our results indicate two candidate markers for sJIA diagnosis and reveal the important roles of platelets and the MAPK pathway in the pathogenesis of sJIA, providing a new perspective for exploring potential molecular targets for sJIA treatment.

## Introduction

Juvenile idiopathic arthritis (JIA) is the most common chronic rheumatologic disease with unknown etiology in childhood. The incidence rate has been reported to vary from 1 to 4 per 1,000 children. Among JIA cases, systemic juvenile idiopathic arthritis (sJIA) accounts for approximately 10–20%, with an incidence rate of 0.4–0.8 cases per 100,000 children (Lee and Schneider, 2018). In general, complications of organ damage and/or macrophage activation syndrome (MAS) will lead to poor prognosis or even death in pediatric sJIA patients (Canny and Mellins, 2017; Lee and Schneider, 2018). Thus, compared with those with other types of JIA, sJIA patients require more intensive systemic treatment and have a higher rate of morbidity. Furthermore, sJIA is characterized by fever, arthritis, skin rash, enlarged lymph nodes, hepatomegaly or splenomegaly, and serositis, manifestations that are not specific, and early diagnosis is difficult because clinical manifestations in the early stage of the disease are often atypical. Therefore, exploration of biomarkers that would help with the early identification and diagnosis of sJIA is urgently needed.

To date, the pathogenesis of sJIA has not been clearly elucidated, and therapeutic strategies are not effective in the management of some sJIA patients. It is believed that the occurrence of sJIA is mainly related to the release of proinflammatory factors from innate immune cells, including TNF-α, IL-1β, and IL-6 (Lee and Schneider, 2018). The biological agents applied are mainly antagonists of IL-1β and IL-6 receptors (Martini, 2019), and inhibitors targeting the JAK-STAT signaling pathway, CTLA or TNF are being evaluated in clinical trials (Ruperto and Martini, 2018). Nevertheless, one-third of patients show only a transient response to treatment, with another one-third having a poor response (Martini, 2019). Hence, there is a pressing need to investigate pathways and small-molecule targets for treatment to improve the prognosis of sJIA.

In recent years, the rapid development of microarray technologies and bioinformatics analysis techniques has contributed to the identification of differentially expressed genes (DEGs) and functional pathways related to JIA. However, there are few bioinformatics analyses of sJIA thus far. Therefore, in the present study, we aimed to explore biomarkers for sJIA early diagnosis, underlying pathogenesis and potential therapeutic targets based on R packages, online tools such as xCell for data processing and qRT-PCR.

## Materials and Methods

### Microarray Data

To identify DEGs significantly related to sJIA, the GSE17590, GSE80060, and GSE112057 gene expression datasets were acquired from the Gene Expression Omnibus database (https://www.ncbi.nlm.nih.gov/geo/) (Quartier et al., 2011; Brachat et al., 2017; Mo et al., 2018). The data were derived from the GPL6106 (Sentrix Human-6 v2 Expression BeadChip), GPL570 (Affymetrix Human Genome U133 Plus 2.0 Array), and GPL11154 (Illumina HiSeq 2000 (*Homo sapiens*)) platforms. The GSE17590 dataset contains 21 healthy individuals and 22 sJIA patients, the GSE80060 dataset 22 healthy individuals and 22 sJIA patients, and the GSE112057 dataset 12 healthy individuals and 26 sJIA patients. The average age of sJIA patients in the GSE17590 dataset is 8.0 years (ranging from 2 to 16 years), and the average age at diagnosis of sJIA patients in the GSE112057 dataset is 6.0 years (ranging from 0.7 to 14 years). In addition, we used the GSE8361 dataset, which is derived from the GPL1291 platform (Hitachisoft AceGene Human Oligo Chip 30K one Chip Version) and contains eight healthy individuals and 51 sJIA patients, for gene validation (Ishikawa et al., 2009). Detailed information on the healthy controls and sJIA patients of GSE17590 and GSE112057 is shown in Supplementary Tables 1A, B. All patients enrolled in GSE80060 were from two trials (ClinicalTrials.gov, NCT00886769 (trial 1) and NCT00889863 (trial 2)) (Ruperto et al., 2012), and control samples were from age-matched, race-matched, and sex-matched healthy subjects. Detailed information on the patients in the GSE8361 dataset is shown in the article of Ishikawa et al. (2009). All gene expression profiles were derived from whole blood samples. For microarray data, probes were converted to official gene symbols according to the platform’s annotation information.

### Data Preprocessing and Quality Assessment

For GSE17590, gene expression data from the matrix file were log2 transformed and subjected to subsequent quantile normalization using the “limma” package in R software (version 4.0.2). For GSE80060, gene expression data were acquired by reading raw cel format files using the “affay” package while performing background correction, after which we standardized the data and removed the batch effects using the “gcrma” and “sva” packages. Finally, for the RNA-seq dataset GSE112057, the raw counts of each sample were acquired from the matrix file, and the count data for each sample were filtered to remove genes with total counts among all samples of less than 20. The remaining data were transformed by vst conversion using the “DESeq2” package.

After data preprocessing, the “arrayQualityMetrics” package, which assesses reproducibility and identifies apparent outlier arrays by providing a report with diagnostic plots, was used to assess the quality of each sample (Kauffmann et al., 2009). Samples were evaluated mainly based on distances between arrays, boxplots and MA plot reports and were removed if outliers were obtained by two or more of the three methods. In addition, 2 samples (GSM3056451 and GSM3056467) in GSE112057 met the exclusion criteria and were excluded from the follow-up analysis. To show the relationships among the remaining samples, a principal component analysis (PCA) plot was generated for each dataset using the “stats” and “ggplot2” packages.

### Differentially Expressed Genes Identification and Analysis

Differential analysis was performed for GSE17590 and GSE80060 using the “limma” package and for GSE112057 using the “DESeq2” package. Genes with | log_2_ (fold change) |≥ log2 (1.5) and adjusted *p* < 0.05 were considered DEGs (Love et al., 2014). The fold changes, significance and gene expression patterns of the DEGs were visualized with volcano plots and heat maps constructed using the “ggplot2” and “pheatmap” packages, respectively. To identify co-DEGs among the three datasets, a Venn diagram was also generated using the online tool Draw Venn Diagram (http://bioinformatics.psb.ugent.be/webtools/Venn/). Overlapping DEGs (co-DEGs) were retained for further analysis.

### Functional Annotation and Pathway Enrichment Analysis

To identify the biological function of co-DEGs, the “clusterProfiler” package was employed for GO annotation and KEGG analysis (Yu et al., 2012), and the online tool Metascape (http://metascape.org/gp/index.html) was employed for comprehensive analysis (Zhou et al., 2019). Terms or pathways with a cutoff of p-value <0.05 were considered significantly enriched. The “clusterProfiler” package was also utilized for GSEA based on gene expression profiles (Subramanian et al., 2005). A default algorithm with 1,000 permutations was applied to calculate enrichment scores and p-values. The gene set size filters were as follows: minimum of 10 and maximum of 500, pvalueCutoff = 0.05, and pAdjustedMethod = “BH”. |NES| >1 and adjusted p-value <0.05 were set as thresholds for identifying significantly enriched gene sets.

### Hub Gene Selection and Analysis

The online tool Search Tool for the Retrieval of Interacting Genes (STRING; http://string-db.org; version 11.0) was used to construct a PPI network for co-DEGs (Szklarczyk et al., 2017; Szklarczyk et al., 2019). The PPI network was visualized using Cytoscape (Shannon et al., 2003), and the molecular complex detection (MCODE) plug-in was used to find clusters of highly interconnected regions in the PPI network (Wang et al., 2015). MCODE score >6, degree cutoff = 2, node score cutoff = 0.2, K-score = 2 and max. depth = 100 were chosen as the cutoff criteria for identifying highly interconnected regions. The cytoHubba plug-in of Cytoscape was employed to identify top hub genes using the MCC algorithm (Chin et al., 2014). The biological functions of the identified hub genes were annotated using the online tool GenCLiP2 (http://ci.smu.edu.cn/GenCLiP2/analysis.php), with thresholds of *p* ≤ 0.05, hit ≥0 and total ≤5,000 (Wang et al., 2014). We incorporated the six hub genes into a HiPlot to identify biomarkers with high sensitivity and specificity (https://hiplot.com.cn/basic/roc).

### Evaluation of Immune Cell Infiltration

To evaluate the abundance of immune infiltrates, we uploaded the gene expression data to xCell (https://xcell.ucsf.edu), which enables analysis of 64 cell types, including immunocytes, stromal cells, stem cells, and other cells (Aran et al., 2017); we only analyzed immune cells in this study. Next, we used HiPlot (https://hiplot.com.cn/basic/cor-heatmap) to draw a correlation heat map to visualize correlations between the 26 types of infiltrating immune cells, and violin plots were drawn with GraphPad Prism software to visualize differences between healthy control (HC) and sJIA patients.

### GSVA

GSVA was performed using the “GSVA” package, a GSE method that estimates variation in pathway activity over a sample population, to analyze datasets from the Molecular Signatures Database (MSigDB, version v7.2) (Subramanian et al., 2005; Liberzon et al., 2011; Hanzelmann et al., 2013; Liberzon et al., 2015). Thresholds were set as |log2 (fold change)| ≥ log2 (1.3) and adjusted p-value <0.05. Correlation analysis between immune cells and significantly enriched KEGG pathways was conducted using SPSS 24.0 software.

### Identification of Small-Molecule Drugs

To identify potential drugs for sJIA treatment, we uploaded 72 upregulated co-DEGs into CMAP (https://clue.io/cmap) (Subramanian et al., 2017). The connectivity score and the enrichment score ranged from 100 to −100, whereby a positive score indicates that a compound can cause or exacerbate the disease state and a negative score that a compound can reduce or even reverse the disease state.

### Patient Recruitment

This study was approved by the Ethics Committee of Children’s Hospital of Chongqing Medical University, and written informed consent was obtained from the participants’ legal guardian/next to kin before enrollment. The diagnosis of sJIA was made according to International League of Associations for Rheumatology (ILAR) Classification Criteria (Petty et al., 2004). We collected whole blood samples from a total of 10 individuals, including five sJIA patients (including one newly diagnosed patient and four treated patients) and five age-matched, race-matched and sex-matched healthy children. Details of the subjects’ characteristics are included in Supplementary Table 1C.

### RNA Extraction and Quantitative Reverse Transcription-Polymerase Chain Reaction PCR

Total RNA was isolated from whole blood from sJIA patients and healthy controls using an RNA extraction kit (Bioteke, Cat#: RP4002) according to the manufacturer’s instructions. The total RNA quality was assessed with a Nanodrop-2000, and complementary DNA (cDNA) was synthesized from 0.8 μg of total RNA using SYBR Premix Ex Taq II (Takara, Cat#: RR820). Reactions were carried out in a total volume of 10 μL, including 5 μL TB Green® Premix Ex Taq™ II (TaKaRa, Cat#: RR820A), 0.5 μL of each specific primer, 3 μL ddH2O, and 1 μL cDNA. Specific primers for each gene and the cycling conditions are listed in Supplementary Table 2.

### Statistical Analyses

All data are expressed as the mean ± standard deviation (SD). Statistical analyses were performed using GraphPad Prism 8. An unpaired Student’s t-test was applied for comparison between healthy individuals and sJIA patients. A p value <0.05 was considered statistically significant.

## Results

### Identification and Analysis of DEGs Between HC and sJIA Patients

**FIGURE 1:**
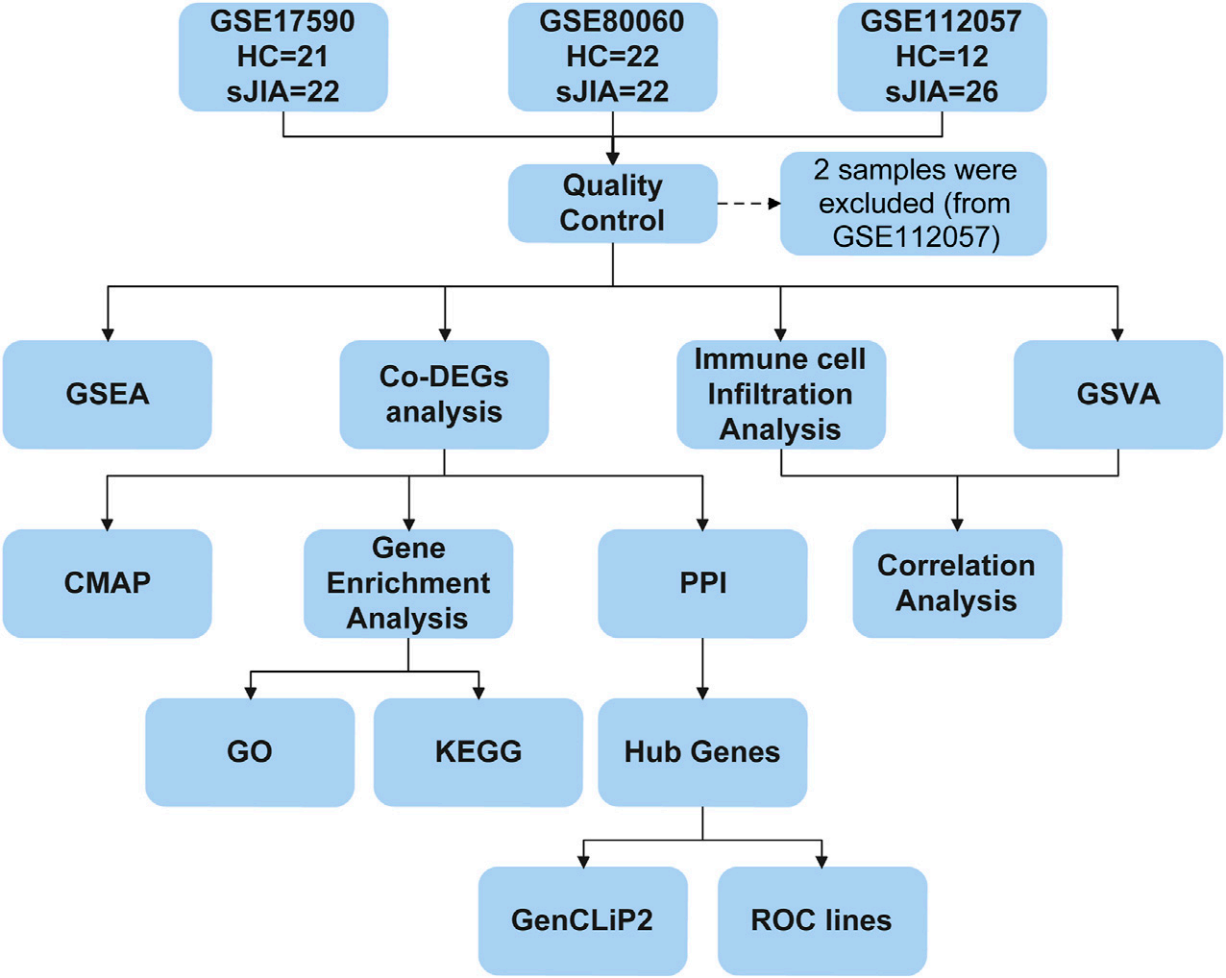
Flow chart of the bioinformatic analysis process. HC, healthy control; sJIA, systemic juvenile idiopathic arthritis; GSEA, gene set enrichment analysis; Co-DEGs, common differentially expressed genes; GSVA, gene set variation analysis; CMAP, connectivity map; PPI, protein-protein interaction; GO, Gene Ontology; KEGG, Kyoto Encyclopedia of Genes and Genomes; ROC, receiver operating characteristic.

**FIGURE 2:**
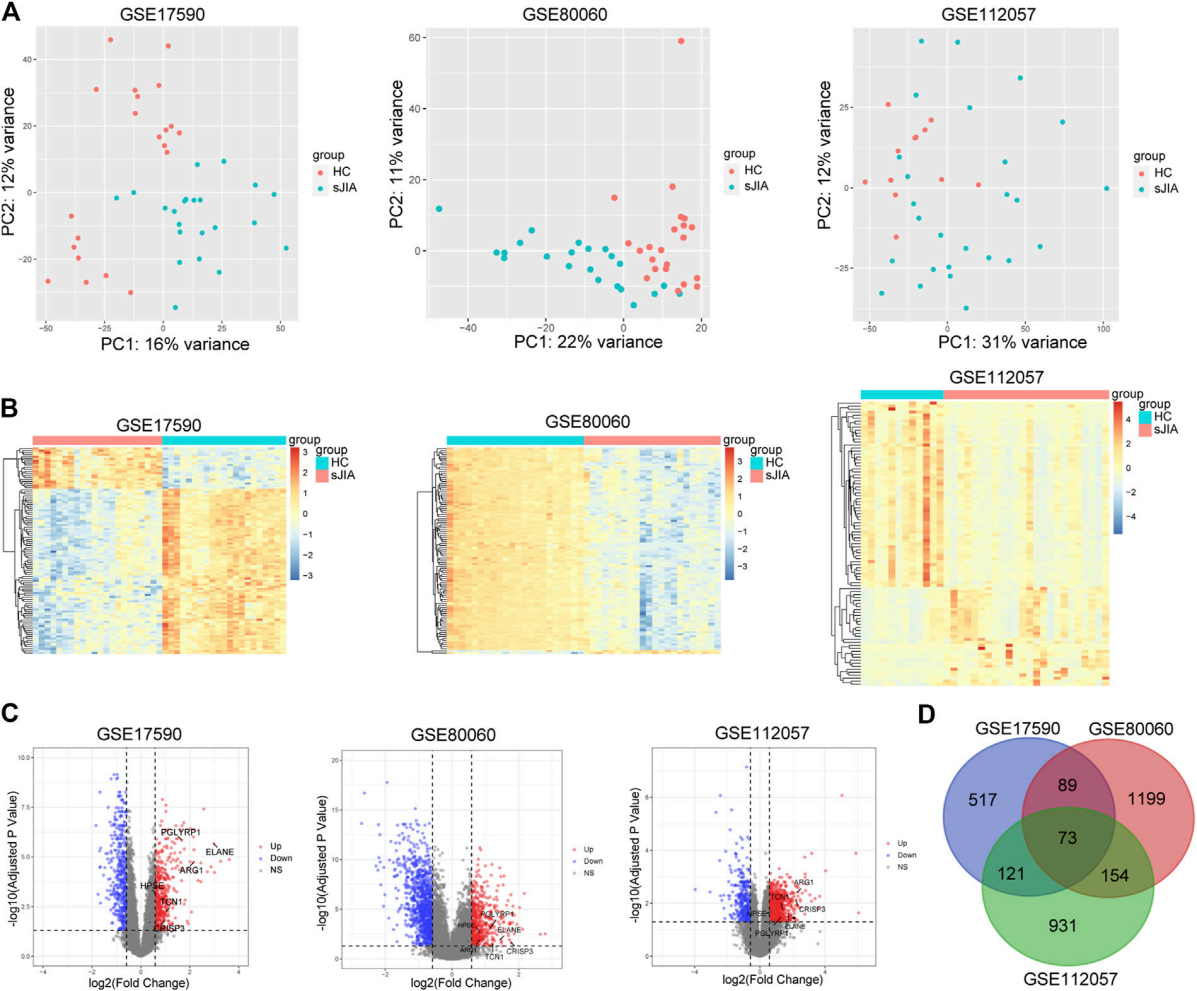
PCA plots and differential analysis of GSE17590, GSE80060, and GSE112057. **(A)** PCA plots of the three datasets. Each point in the scatter plots represents a GSM sample. **(B)** Heat maps of the top 100 DEGs between HC and sJIA patients. **(C)** Volcano plots of the DEGs between HC and sJIA patients. The blue dots represent significantly downregulated genes the red dots significantly upregulated genes in sJIA patients; hub genes are marked. **(D)** Venn diagram showing overlapping DEGs of the three datasets. PCA, principal component analysis; DEGs, differentially expressed genes; HC, healthy control; sJIA, systemic juvenile idiopathic arthritis.

The study workflows are shown in Figure 1. After preprocessing, the distribution of expression data across samples in each dataset was consistent; therefore, the samples of each dataset were comparable, as shown in Supplementary Figure 1. GSM samples in the three datasets were significantly different between HCs and sJIA patients, as depicted in Figure 2A. We identified 857 (444 upregulated and 413 downregulated) DEGs in the GSE17590 series, 1,515 (522 upregulated and 993 downregulated) DEGs in the GSE80060 series, and 1,279 (932 upregulated and 347 downregulated) DEGs in the GSE112057 series, as illustrated in heat maps and volcano plots in Figures 2B,C. In addition, after transforming entrez ID into gene symbols, we searched for co-DEGs of these three datasets and identified 73 (72 upregulated and 1 downregulated), as indicated in the Venn diagram in Figure 2D.

### Functional Annotation of DEGs and GSEA

**FIGURE 3:**
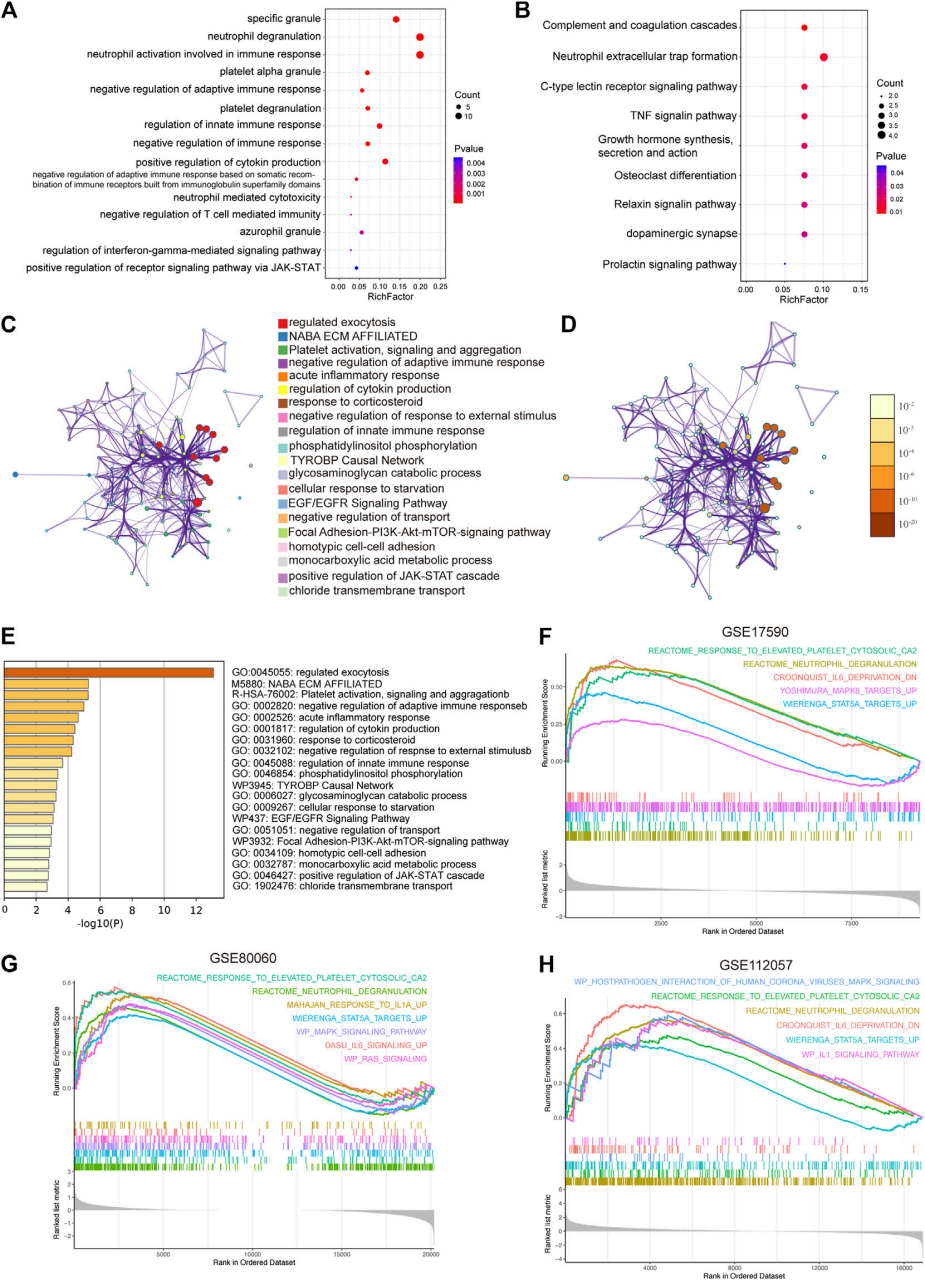
Functional enrichment analysis. **(A)** Significantly enriched Gene Ontology terms of co-DEGs. **(B)** Significantly enriched Kyoto Encyclopedia of Genes and Genomes pathways of co-DEGs. **(C)** Network of representative enriched terms of co-DEGs. Each node represents an enriched term and is colored by cluster identity. **(D)** The same enrichment network with its nodes colored by *p* values. **(E)** Top 20 pathways related to co-DEGs based on comprehensive enrichment analysis using the GO, KEGG, and Reactome databases. **(F-H)** Significantly enriched gene set enrichment analysis pathways between HCs and sJIA patients in the GSE17590, GSE80060, and GSE112057 datasets. Co-DEGs, common differentially expressed genes.

The results of GO, KEGG and Metascape analyses revealed enhanced function of neutrophils and platelets in sJIA and a negative regulation of T cell-mediated immunity (Figures 3A–E). In addition, we conducted GSEA to identify biological differences between HC and sJIA patients, and differential gene thresholds did not need to be set for the GO and KEGG analyses (Figures 3F–H). GSEA revealed elevated neutrophil and platelet granules and upregulation of the IL-1, IL-6, RAS, MAPK, and STAT5 pathways in sJIA. Moreover, enrichment analysis indicated enhanced function of neutrophils and platelets, negative regulation of T cell-mediated immunity, and upregulation of the MAPK and JAK-STAT pathways in sJIA patients.

### Hub Gene Selection and Biological Function Analysis

**FIGURE 4:**
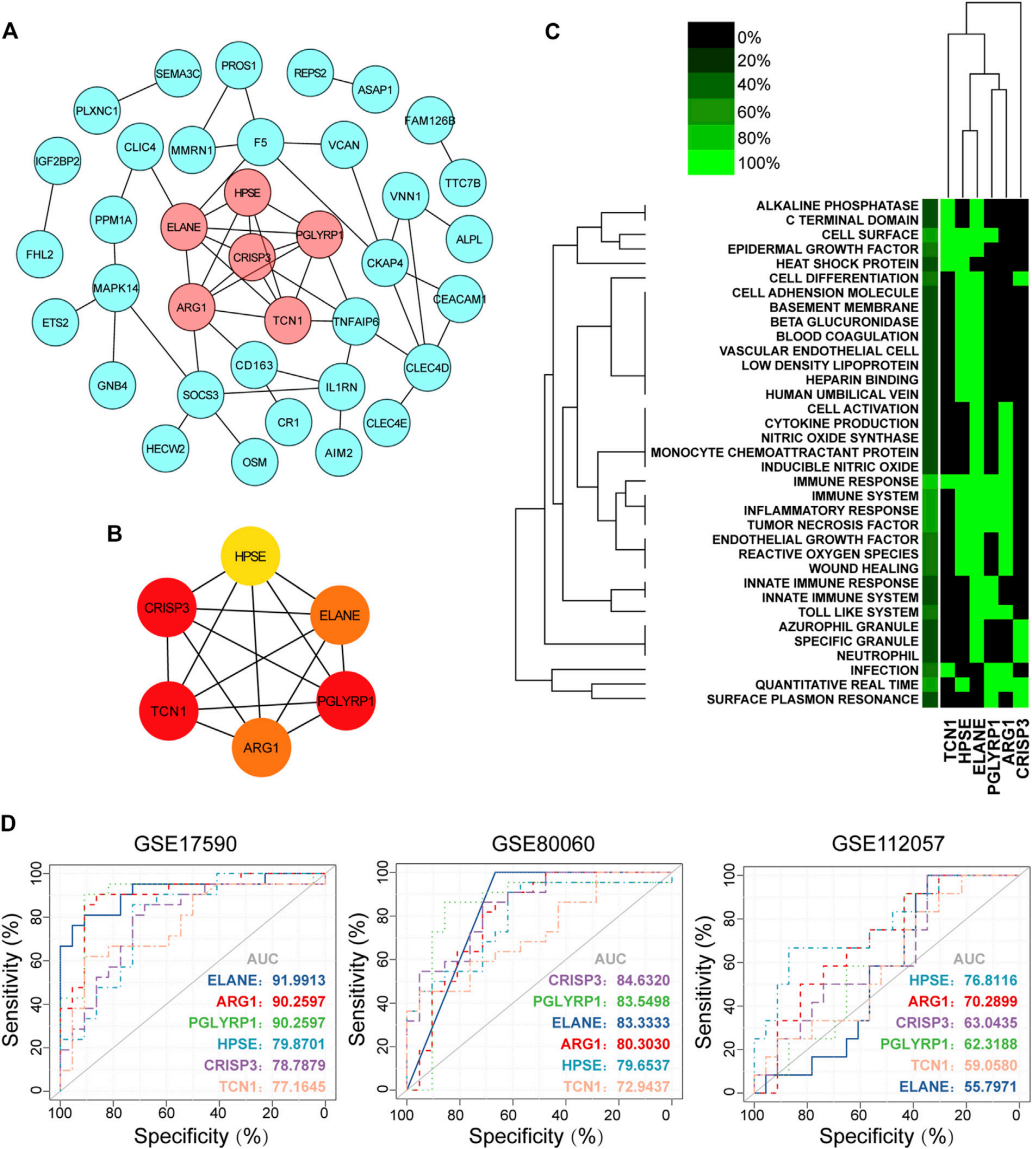
Hub gene identification, functional annotation and diagnosis prediction analysis**. (A)** PPI network of co-DEGs; nodes in pink denote the highly interconnected region identified by “MCODE”. **(B)** Networks of the six hub genes identified by “cytoHubba”. The yellow to red color scale denotes the *p* value calculated by the MCC method. **(C)** Functional heat map of the six hub genes based on literature mining. Green indicates that a gene term has been reported to have a positive association, and black indicates that the gene term has not been reported to have an association. In the left-most lane, green indicates the proportion of genes associated with the term. **(D)** Diagnostic utility of the six hub genes in the three datasets. PPI, protein-protein interaction; co-DEGs, common differentially expressed genes; ROC, receiver operating characteristic; AUC, area under the ROC curve.

Most co-DEGs were upregulated genes, with only one downregulated gene. Therefore, in the ensuing analysis, we focused on upregulated genes in sJIA. We constructed a PPI network to analyze hub genes, and six were identified: HPSE, CRISP3, TCN1, PGLYRP1, ARG1, and ELANE. These hub genes were surrounded by 31 gene nodes significantly related to the DEGs (Figures 4A,B). In addition, GenCLiP2 was used to analyze the biological functions of the hub genes, as shown in Figure 4C, and the innate immune response, especially enhanced function of neutrophils and platelets, was obviously related to the hub genes. The iRegulon plugin of Cytoscape was employed for transcription factor analysis, with a threshold of NES ≥10; five transcription factors were identified, RUNX2, FOXD1, RUNX1, FOXP3, and TGIF1, among the hub genes.

### Analysis of Hub Gene Diagnostic Significance

To explore the diagnostic value of co-DEGs for sJIA, ROC analysis was used to explore sensitivity and specificity values. The results showed that ELANE, ARG1, and PGLYRP1 had the best diagnostic value for distinguishing sJIA patients from HCs in the three datasets **(** Figure 4D). Furthermore, ROC curve analysis of the model in the GSE17590 training set (AUC = 0.919913, 0902597, and 0.902597 for ELANE, ARG1, and PGLYRP1, respectively) and GSE80060 (AUC = 0.833333, 0.803030, and 0.835498 for ELANE, ARG1, and PGLYRP1, respectively) showed the good predictive value of the hub genes for sJIA.

### Immune Cell Infiltration Analysis

**FIGURE 5:**
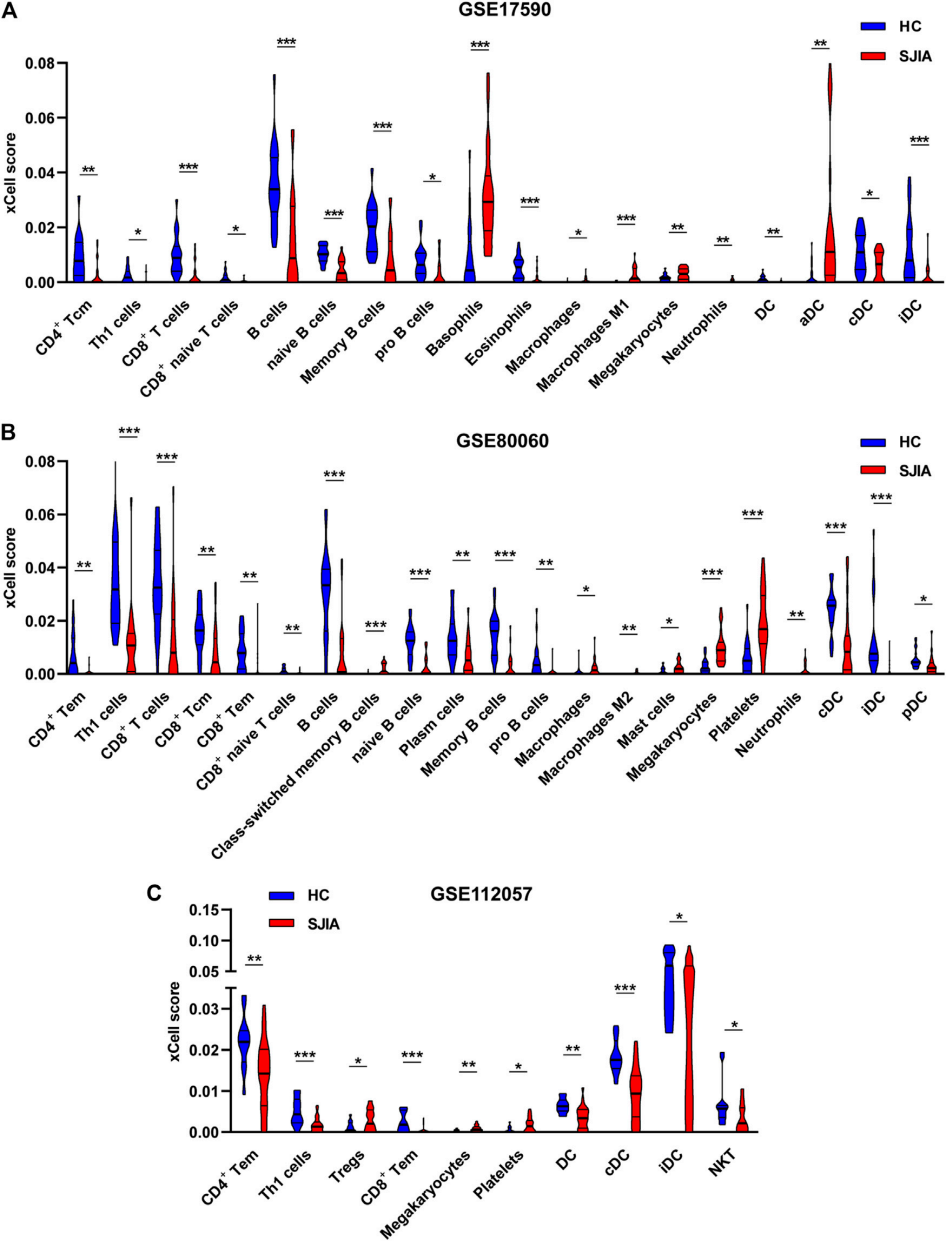
Immune cell infiltration analysis of the three datasets. The **(A)** GSE17590, **(B)** GSE80060, and **(C)** GSE112057 datasets were analyzed to determine differences in immune cell infiltration between HCs and sJIA patients. HC, healthy control; sJIA, systemic juvenile idiopathic arthritis.

**FIGURE 6:**
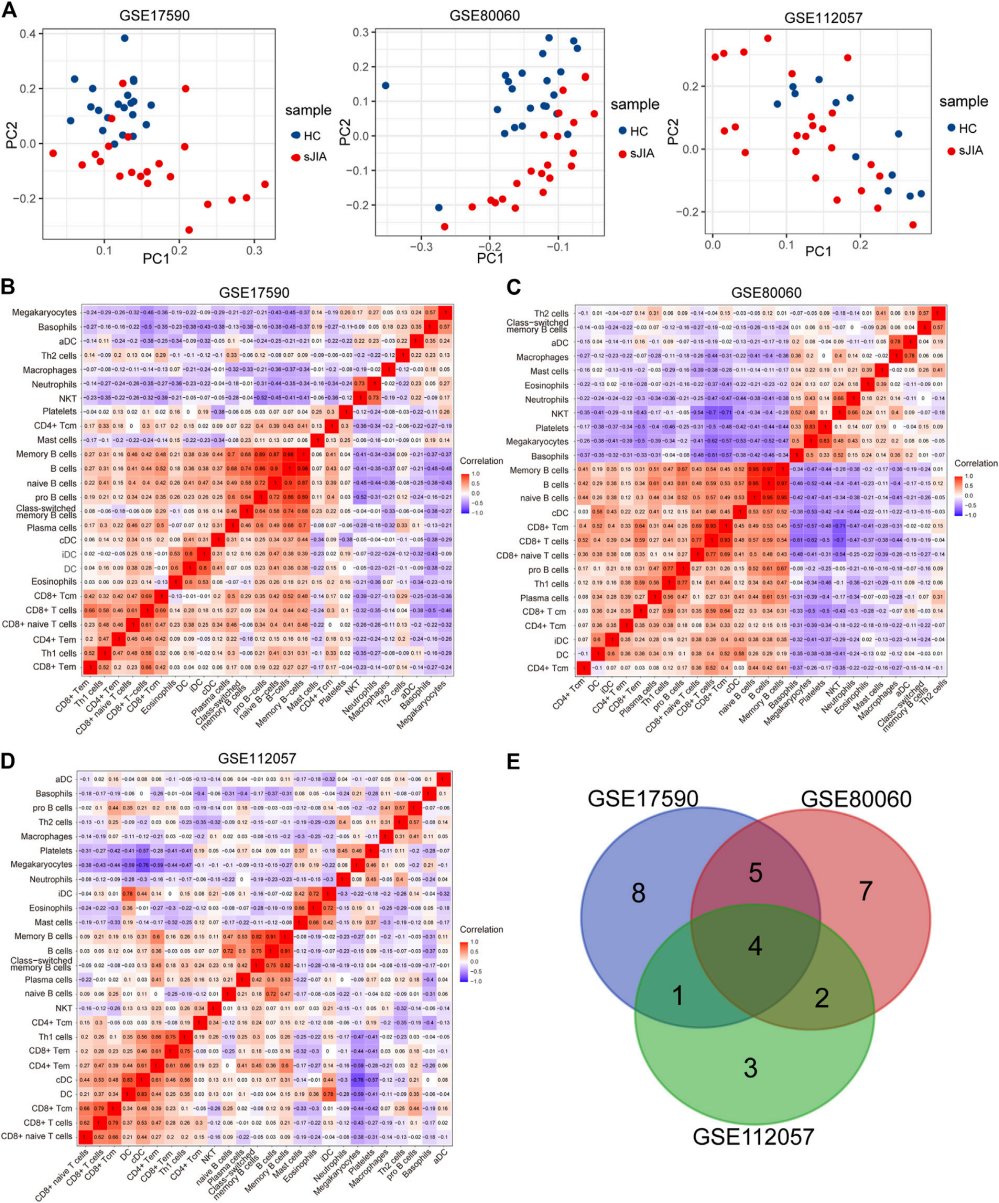
Analysis of immune cells between HCs and sJIA patients. **(A)** PCA using GSE17590, GSE80060, and GSE112057 to uncover differences in immune phenotypes between HC and sJIA patients. **(B-D)** Correlation heat maps of immune cells in the three datasets. **(E)** Venn diagram showing overlapping immune cells between HCs and sJIA patients. PCA, principal component analysis; HC, healthy control; sJIA, systemic juvenile idiopathic arthritis.

To determine which cell types may be involved in the pathogenesis of sJIA, we used the online tool xCell (https://xcell.ucsf.edu/), which generates cell type enrichment scores based on gene expression data. The cell types associated with sJIA in the three datasets were notably different, as illustrated in Figures 5A–C. Compared with HCs, patients with sJIA in all datasets showed less infiltration of Th1 cells and more infiltration of megakaryocytes. In GSE17590 and GSE80060, neutrophil infiltration was increased in sJIA; in GSE80060 and GSE112057, platelets showed increased infiltration in sJIA. In addition, as indicated in the PCA plot, compared with HCs, sJIA patients in the three datasets showed obvious group-bias clustering and individual differences in the proportions of immune cells (Figure 6A). The correlation heat map of immune cells indicated that platelets had a negative correlation with conventional dendritic cells (cDCs), Th1 cells had a positive correlation with CD8 effector memory T (Tem) cells, and memory B cells had significant positive correlations with B cells and B cell subsets in the three datasets. Megakaryocytes exhibited a positive correlation with basophils and neutrophils with natural killer T (NKT) cells in GSE17590 and GSE80060 (Figures 6B–D). In total, there were four common immune cell types with significant differences between patients with sJIA and HCs in all three datasets: T helper 1 (Th1) cells, immature dendritic cells (iDCs), cDCs, and megakaryocytes. Another five common immune cell types showed significant differences between sJIA patients and HCs in GSE17590 and GSE80060: neutrophils, naïve B cells, pro-B cells, naïve CD8^+^ T cells, and macrophages (Figure 6E).

### GSVA and Correlation Analysis Between Significant Pathways and Immune Cells

**FIGURE 7:**
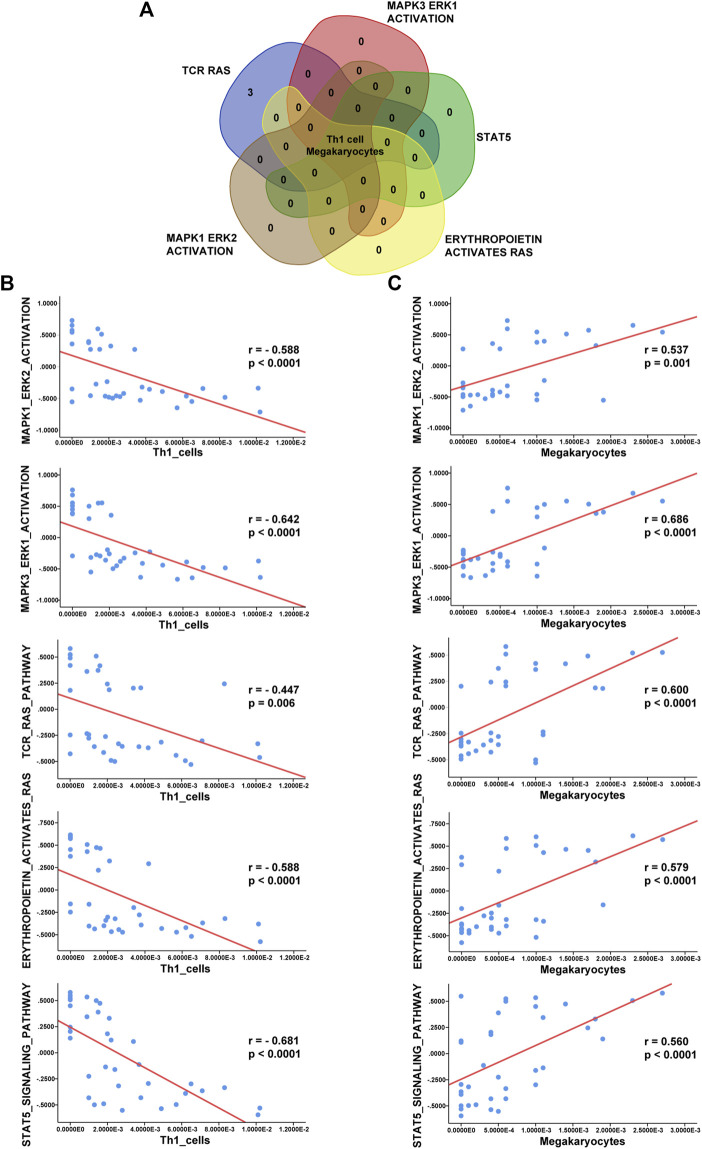
Correlation analysis between immune cells and enriched pathways. **(A)** Venn diagram showing that Th1 cells and megakaryocytes correlate with the terms “MAPK3 ERK1 ACTIVATION”, “MAPK1 ERK2 ACTIVATION”, “TCR RAS”, “ERYTHROPOIETIN ACTIVATES RAS”, and “STAT5”. **(B)** Analysis of correlations of Th1 cells with these five enriched terms in GSE112057. **(C)** Analysis of correlations of megakaryocytes with these five enriched terms in GSE112057.

According to the Venn diagram in Figure 7A **,** Th1 cells and megakaryocytes are common infiltrating immune cells across the three datasets. To evaluate associations between the MAPK, RAS, and JAK-STAT pathways and immune cell infiltration, we used Spearman correlation analysis to determine correlations of the GSVA enrichment scores of these signaling pathways with the xCell scores of immune cells. In addition, we determined correlations between Th1 cells and megakaryocytes and the terms “MAPK1 ERK2 ACTIVATION”, “MAPK3 ERK1 ACTIVATION”, “TCR RAS”, “ERYTHROPOIETIN ACTIVATES RAS”, and “STAT5” based on the GSE112057 dataset **(** Figures 7B,C). Additional scatter plots displaying the correlations in GSE17590 and GSE80060 are provided in Supplementary Figures 2, 3. Overall, we observed significant negative correlations between Th1 cells and the above pathways and positive correlations between megakaryocytes and the above pathways, indicating close interactions between these two immune cell types and the MAPK and STAT pathways.

### Identification of Related Active Small Molecules

**FIGURE 8:**
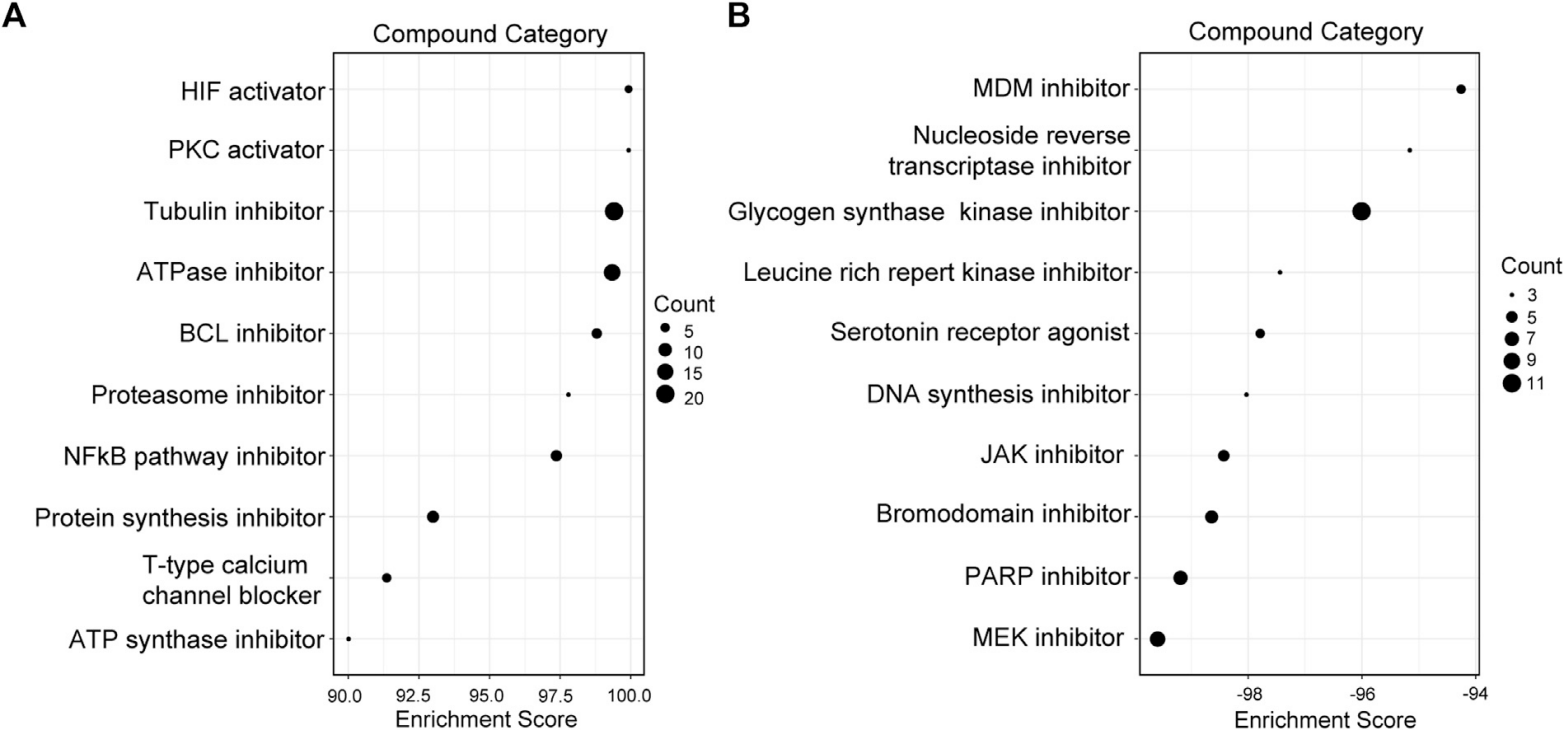
Screening of candidate compounds for sJIA patients. **(A)** Compound perturbations with enrichment scores above 90. **(B)** Compound perturbations with enrichment scores below -90. sJIA, systemic juvenile idiopathic arthritis.

Compound categories with positive enrichment scores and negative enrichment scores identified via CMAP analysis are presented in Figures 8A,B. Kinases of the MAPK family comprise three subfamilies: extracellular signal-regulated kinases (ERKs), c-Jun N-terminal kinases (JNKs), and p38 mitogen-activated protein kinases. Mitogen-activated protein kinase kinase (MEK) is a kinase that phosphorylates ERK1/2. When MEK is inhibited by a MEK inhibitor, activation of downstream ERK1/2 is also inhibited, blocking cell proliferation and inducing apoptosis (Shi and Sun, 2018). Our results showed the MEK inhibitor to be the most significant compound perturbation that might reverse sJIA, as illustrated in Figure 8B. Based on the top 20 small molecules (ranked by connectivity score) with targets and mechanisms of action (MOAs), most are MEK inhibitors or kinase inhibitors of the MAPK family, as listed in Table 1. These small-molecule candidates target the co-DEGs in sJIA, affecting gene expression and function. These results indicate that MEK inhibitors and some kinase inhibitors of the MAPK family are candidate agents for the treatment of sJIA.

**TABLE 1:** The 20 most significant small-molecule drugs with negative connectivity scores (suggesting that they can reverse sJIA).

Rank	Name	Score	Target	MOA
1	JAK3-inhibitor-I*	-99.89	JAK3	JAK inhibitor
2	Cyclo-[Arg-Gly-Asp-D-Phe-Val]	-99.89	ITGAV, ITGB3	Integrin antagonist
3	Phenazone	-99.89	PTGS1, PTGS2	Cyclooxygenase inhibitor
4	SC-68376*	-99.82	MAPK14	p38 MAPK inhibitor
5	PD-123319	-99.77	AGTR2	Angiotensin receptor antagonist
6	CG-930*	-99.72	MAPK8	JNK inhibitor
7	Lapatinib	-99.7	EGFR, ERBB2, CYP3A5	EGFR inhibitor, ErbB2 inhibitor
8	Tipifarnib	-99.68	FNTA, FNTB	Farnesyltransferase inhibitor
9	PKCbeta-inhibitor	-99.68	PRKCB	PKC inhibitor
10	MK-1775	-99.61	WEE1	WEE1 kinase inhibitor
11	T-0070907	-99.58	PPARG	PPAR receptor antagonist
12	QL-XI-92	-99.44	DDR1	DDR1 inhibitor
13	AS-703026*	-99.22	MAP2K1, MAP2K2	MEK inhibitor
14	PD-98059*	-99.12	MAP2K1, MAP2K2, MAPK1, MAPK11, MAPK12, MAPK14, MAPK3, MAPK8	MEK inhibitor, MAP kinase inhibitor
15	Selumetinib*	-99.05	MAP2K1, MAP2K2	MEK inhibitor
16	Palonosetron	-99.01	HTR3A	Serotonin receptor antagonist
17	Cisapride	-98.96	HTR4, KCNH2, CYP2B6, CYP2C8, CYP3A5, CYP3A7	Serotonin receptor agonist
18	Terazosin	-98.91	ADRA1D, ADRA1A, ADRA1B, KCNH2, KCNH6,	Adrenergic receptor antagonist
19	PD-0325901*	-98.91	MAP2K1, MAP2K2	MEK inhibitor, MAP kinase inhibitor
20	LY-303511	-98.87	CSNK2A1, CSNK2A2, CSNK2B, MTOR	Casein kinase inhibitor, MTOR inhibitor, PI3K inhibitor

A small molecule with * indicates that its MOA is a MEK inhibitor and/or MAPK inhibitor or JAK inhibitor.

MOA, mechanism of action.

### Validation of Hub Gene Expression

**FIGURE 9:**
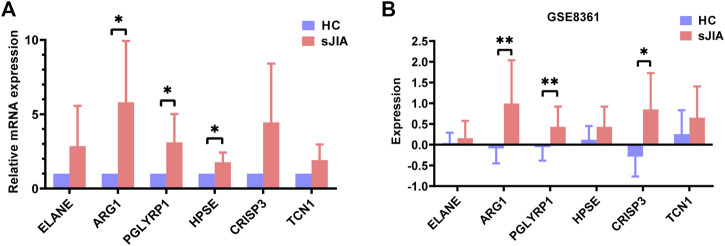
Validation of ELANE, ARG1, PGLYRP1, HPSE, CRISP3, and TCN1 expression in blood samples from healthy controls and sJIA patients. **(A)** qRT-PCR validation of six hub genes. **(B)** Expression levels validation in the GSE8361 dataset. sJIA, systemic juvenile idiopathic arthritis.

To verify the accuracy of the transcriptomic data, six hub genes of HPSE, CRISP3, TCN1, PGLYRP1, ARG1, and ELANE were validated by qRT-PCR in five healthy individuals and five sJIA patients (Figure 9A). The mRNA levels of ARG1, PGLYRP1, and HPSE were significantly upregulated in sJIA patients compared with healthy controls (Figure 9A). Similar results were obtained by comparing the expression levels of the hub genes in the GSE8361 dataset (51 sJIA patients and eight healthy controls), revealing upregulation of ARG1, PGLYRP1 and CRISP3 by microarray (Figure 9B).

## Discussion

JIA is the most common type of arthritis in children, and sJIA is the most serious type of JIA. To identify markers that are conducive to early diagnosis, identify pathways related to pathogenesis and reveal small molecules with potential therapeutic effects, we performed bioinformatic analysis of the GSE17590, GSE112057, and GSE80060 datasets using a variety of tools. We found six hub genes and identified by ROC curve analysis that ARG1, PGLYRP1, and ELANE have high diagnostic sensitivity and specificity. Through GO and KEGG analyses and GSEA, we found that enhanced functions of neutrophils and platelets and upregulation of the MAPK pathway are involved in the pathogenesis of sJIA; according to the immune infiltration analysis, megakaryocytes are positively related to the MAPK pathway. Moreover, we used CMAP analysis to identify targeted pathways and potential small-molecule compounds for the treatment of sJIA.

For experimental validation, we performed qRT-PCR for 10 individuals, including five sJIA patients (including one newly diagnosed patient and four treated patients) and five age-matched, race-matched and sex-matched healthy children, to detect relative expression levels of the six identified hub genes. The results showed the mRNA levels of ARG1, PGLYRP1, and HPSE to be significantly upregulated in sJIA patients compared with healthy controls, which is consistent with the results of the bioinformatic analysis. Although ELANE, TCN1, and CRISP3 showed no significant changes between sJIA patients and healthy controls, higher average relative mRNA levels were observed for all three genes (Figure 9A). We speculate that the nonsignificance may be attributed to the limited sample size. Despite efforts to obtain more blood samples from sJIA patients, the sample size was limited, especially for untreated acute sJIA samples, which were also reported by Henderson et al. to be difficult to obtain (Henderson et al., 2020). Fortunately, Mo et al. revealed that gene expression patterns may not be significantly altered after treatment with medication (Mo et al., 2018); thus, it was reasonable to combine the treated and untreated patients in our validation experiments. Moreover, we only detected mRNA expression levels, which cannot fully reflect protein levels due to the various processes involved in expression, including transcription, posttranscriptional modification, translation, and posttranslational modification. We did not detect protein levels at that time because of the insufficient number of cases and the limited amount of blood collected from each child (namely, ethical issues).

In this study, ARG1 and PGLYRP1 were identified as potential diagnostic markers for sJIA. The arginase 1 (ARG1) gene encodes arginase type I, which metabolizes L-arginine to L-ornithine and urea. In human, arginase one is constitutively expressed in polymorphonuclear neutrophils, and acts as a key regulator of innate immunity and adaptive immunity (Panfili et al., 2020). It is liberated from neutrophils in some inflammatory diseases such as glomerular acute immune complex inflammation, and it reduces the L-arginine bioavailability and downregulates the levels of NO through substrate competition, which in turn suppresses immune responses (Waddington et al., 1998; Munder, 2009; Chang et al., 2020; Sim et al., 2021). In the present study, ARG1 was found to be upregulated in sJIA patients, which may be attributed to the chronic arthritis of sJIA, since ARG1 is supposed to be an anti-inflammatory factor. Although we have known that arginase one mainly acts as a key regulator of extracellular arginine metabolism (Munder et al., 2006; Jacobsen et al., 2007; Rotondo et al., 2011), it remains unclear whether and how ARG1 affects neutrophils. In addition, no literature is available on the relationship between ARG1 and platelets. As for the peptidoglycan recognition protein 1 (PGLYRP1), it is firstly reported in 1998 (Kang et al., 1998), and is highly expressed in polymorphonuclear leukocytes (Dziarski and Gupta, 2006). It plays a proinflammatory role through the activation of innate immune mechanism, and has been reported to be upregulated in rheumatoid arthritis patients (Dziarski and Gupta, 2010; Luo et al., 2019). However, no study has ever reported the relationship between PGLYRP1 and sJIA, and the role of PGLYRP1 in neutrophils and platelets also remains poorly understood.

Our study indicated obvious increases in platelet granules and megakaryocytes in sJIA. Megakaryocytes mature in the bone marrow, enter the peripheral circulation by penetrating the venous sinus endothelium and release platelets. Thus, increased numbers of megakaryocytes can result in an increased number of platelets released, which is consistent with the results of previous studies showing elevated platelets in sJIA (Wagner-Weiner, 2015; Schulert et al., 2018; Wang et al., 2019). In turn, an increased number of platelets secrete increased levels of granules, including cytokines and chemokines such as IL-1β, thus recruiting neutrophils and inducing an autoinflammatory response (Rolfes et al., 2020). Hence, an increased number of platelets is closely related to a severe inflammatory phenotype and prognosis in early-onset sJIA (Russo and Katsicas, 2013; Eloseily et al., 2020). We also found enhanced neutrophil function in sJIA, which is consistent with previous studies. High neutrophil counts, abundant neutrophil granules and increased levels of related genes (ELANE, MPO, and MMP8) are observed in JIA (Ramanathan et al., 2018; Ter Haar et al., 2019). Further studies are required to determine the specific mechanisms of neutrophils and platelets in sJIA.

Previous literature has confirmed that the innate immune response plays a more important role than the adaptive immune response in the pathogenesis of sJIA. In our study, immune infiltration analysis indicated decreased Th1 cell infiltration in sJIA, and GO and KEGG analyses and GSEA revealed downregulation of the adaptive immune response. In fact, there is currently no consensus with regard to the types of changes in T cells and adaptive immune responses that occur in sJIA. This lack of consensus may be explained by the different stages of disease included in each study, suggesting varied disease activity among the patients studied. Overall, the changes occurring in CD4^+^ T cells in sJIA reported in different studies are inconsistent, with some studies reporting increases (Omoyinmi et al., 2012), some that levels do not differ significantly from those in HCs (Macaubas et al., 2010), and some that they are decreased in proportion and in size in children with active sJIA compared with normal children (Barnes et al., 2009; Mellins et al., 2011; Cai et al., 2019). Further studies are needed to validate the phenotype of T cells and the adaptive immune response status in different stages of sJIA.

Until recently, the pathogenesis of sJIA has remained unclear, though it has been widely reported that the IL-1 and IL-6 signaling pathways and JAK-STAT pathway are markedly upregulated (Ogilvie et al., 2007; Jiang et al., 2020). Our study showed that the mitogen-activated protein kinase (MAPK) pathway, which is reported to regulate inflammation, cell proliferation, cell differentiation, cell survival and apoptosis, is upregulated in sJIA (Sun et al., 2015). Moreover, CMAP results indicated that MEK inhibitors and some kinase inhibitors of the MAPK family are candidate agents for the treatment of sJIA. In fact, MAPK pathway upregulation is rarely reported in sJIA patients, even though it plays vital roles in the pathogenesis of inflammatory diseases, such as rheumatoid arthritis and Crohn’s disease (Coulthard et al., 2009; Yang et al., 2018), which suggests that the MAPK pathway may be related to sJIA. We speculate that the possible mechanism might be as follows. In sJIA, after increased IL-6 binds to the receptor gp130, SHP-2 is recruited to the gp130 phosphorylated Tyr759 residue, phosphorylated by JAK, and then acts on Grb2 to promote Ras GDP/GTP exchange. Ras and Raf bind and activate Raf, Raf phosphorylation activates MEK, and further phosphorylation activates ERK1/ERK2 (Pope, 2002; Mihara et al., 2012), thereby promoting proliferation and inhibiting apoptosis of inflammatory cells, which might contribute to pathogenesis.

In conclusion, six hub genes were identified in this study, of which ARG1 and PGLYRP1 are potential biomarkers for the early diagnosis of sJIA. Moreover, the present study revealed the important roles of platelets and the MAPK pathway in the pathogenesis of sJIA, providing a new perspective for exploring potential molecular targets for the treatment of sJIA. Although we validated upregulation of ARG1 and PGLYRP1 by qRT-PCR in sJIA patients, proteins and pathways were not verified in the present study. As changes in mRNA do not completely reflect changes in protein levels or pathway functions, further studies are needed to explore the changes in the identified genes at the protein level and from a functional point of view. In addition, due to the limited amount of blood collected from each child (due to ethical issues), it was difficult to conduct extensive mechanistic studies in a limited time, which is a major limitation. Studies with large sample sizes and further mechanistic analysis are needed to confirm our findings.

## Data Availability

The datasets presented in this study can be found in online repositories. The names of the repository/repositories and accession number(s) can be found in the article/Supplementary Material.
