# Supplementary material for: Identification of Key Biomarkers and Immune Infiltration in Systemic Juvenile Idiopathic Arthritis by Integrated Bioinformatic Analysis
Source: Front Mol Biosci. 2021 Jul 14;8:681526. doi: 10.3389/fmolb.2021.681526 (PMC8316978; doi:10.3389/fmolb.2021.681526)
Supplement: Supplementary file 1 [file Table1.docx]

**Supplementary Table 1A. Characteristics of patients included in GSE17590**

| **Sample_ID** | **Gender** | **Age (years)** | **Diagnosis** | **Tissue** |
| --- | --- | --- | --- | --- |
| GSM438137 | Female | 5 | Control | Whole Blood |
| GSM438143 | Female | 9 | Control | Whole Blood |
| GSM438152 | Female | 4 | Control | Whole Blood |
| GSM438157 | Female | 11 | Control | Whole Blood |
| GSM438159 | Female | 10 | Control | Whole Blood |
| GSM438170 | Female | 16 | Control | Whole Blood |
| GSM438174 | Male | 14 | Control | Whole Blood |
| GSM438179 | Female | 19 | Control | Whole Blood |
| GSM438186 | Male | 0.8 | Control | Whole Blood |
| GSM438191 | Female | 4 | Control | Whole Blood |
| GSM438198 | Female | 15 | Control | Whole Blood |
| GSM438199 | Female | 14 | Control | Whole Blood |
| GSM438203 | Female | 19 | Control | Whole Blood |
| GSM438204 | Male | 14 | Control | Whole Blood |
| GSM438208 | Male | 9 | Control | Whole Blood |
| GSM438212 | Female | 10 | Control | Whole Blood |
| GSM438213 | Female | 8 | Control | Whole Blood |
| GSM438217 | Male | 13 | Control | Whole Blood |
| GSM438218 | Male | 12 | Control | Whole Blood |
| GSM438219 | Male | 6 | Control | Whole Blood |
| GSM438220 | Female | 9 | Control | Whole Blood |
| GSM438138 | Male | 9 | Systemic JIA | Whole Blood |
| GSM438142 | Female | 2 | Systemic JIA | Whole Blood |
| GSM438145 | Female | 7 | Systemic JIA | Whole Blood |
| GSM438147 | Female | 15 | Systemic JIA | Whole Blood |
| GSM438150 | Male | 10 | Systemic JIA | Whole Blood |
| GSM438154 | Male | 5 | Systemic JIA | Whole Blood |
| GSM438158 | Female | 4 | Systemic JIA | Whole Blood |
| GSM438162 | Male | 7 | Systemic JIA | Whole Blood |
| GSM438166 | Female | 7 | Systemic JIA | Whole Blood |
| GSM438168 | Female | 4 | Systemic JIA | Whole Blood |
| GSM438172 | Female | 15 | Systemic JIA | Whole Blood |
| GSM438176 | Female | 7 | Systemic JIA | Whole Blood |
| GSM438180 | Female | 7 | Systemic JIA | Whole Blood |
| GSM438183 | Male | 14 | Systemic JIA | Whole Blood |
| GSM438187 | Female | 10 | Systemic JIA | Whole Blood |
| GSM438189 | Female | 10 | Systemic JIA | Whole Blood |
| GSM438194 | Male | 4 | Systemic JIA | Whole Blood |
| GSM438195 | Male | 3 | Systemic JIA | Whole Blood |
| GSM438201 | Male | 3 | Systemic JIA | Whole Blood |
| GSM438205 | Female | 16 | Systemic JIA | Whole Blood |
| GSM438209 | Female | 7 | Systemic JIA | Whole Blood |
| GSM438214 | Female | 11 | Systemic JIA | Whole Blood |

**Supplementary Table 1B. Characteristics of patients included in GSE112057**

| **Sample_ID** | **Gender** | **Age (years)** | **Ancestry** | **Diagnosis** | **Tissue** |
| --- | --- | --- | --- | --- | --- |
| GSM3056569 | NA | NA | NA | Control | Whole Blood |
| GSM3056570 | NA | NA | NA | Control | Whole Blood |
| GSM3056571 | NA | NA | NA | Control | Whole Blood |
| GSM3056572 | NA | NA | NA | Control | Whole Blood |
| GSM3056573 | NA | NA | NA | Control | Whole Blood |
| GSM3056574 | NA | NA | NA | Control | Whole Blood |
| GSM3056575 | NA | NA | NA | Control | Whole Blood |
| GSM3056576 | NA | NA | NA | Control | Whole Blood |
| GSM3056577 | NA | NA | NA | Control | Whole Blood |
| GSM3056578 | NA | NA | NA | Control | Whole Blood |
| GSM3056579 | NA | NA | NA | Control | Whole Blood |
| GSM3056580 | NA | NA | NA | Control | Whole Blood |
| GSM3056406 | Female | 1 | Black | Systemic JIA | Whole Blood |
| GSM3056407 | Female | 1.5 | Black | Systemic JIA | Whole Blood |
| GSM3056417 | Male | 10 | White | Systemic JIA | Whole Blood |
| GSM3056418 | Female | 1.5 | White | Systemic JIA | Whole Blood |
| GSM3056421 | Male | 1 | White | Systemic JIA | Whole Blood |
| GSM3056451 | Male | 1 | White | Systemic JIA | Whole Blood |
| GSM3056467 | Male | 6 | White | Systemic JIA | Whole Blood |
| GSM3056469 | Male | 1 | White | Systemic JIA | Whole Blood |
| GSM3056470 | Female | 10 | White | Systemic JIA | Whole Blood |
| GSM3056488 | Male | 3 | Black | Systemic JIA | Whole Blood |
| GSM3056495 | Male | 14 | White | Systemic JIA | Whole Blood |
| GSM3056499 | Male | 2.5 | White | Systemic JIA | Whole Blood |
| GSM3056503 | Female | 6 | Black | Systemic JIA | Whole Blood |
| GSM3056514 | Female | 0.7 | White | Systemic JIA | Whole Blood |
| GSM3056515 | Male | 13 | Black | Systemic JIA | Whole Blood |
| GSM3056517 | Female | 14 | Black | Systemic JIA | Whole Blood |
| GSM3056518 | Female | 7 | Black | Systemic JIA | Whole Blood |
| GSM3056529 | Female | 4 | White | Systemic JIA | Whole Blood |
| GSM3056536 | Female | 8 | White | Systemic JIA | Whole Blood |
| GSM3056550 | Male | 10 | White | Systemic JIA | Whole Blood |
| GSM3056561 | Male | 2 | White | Systemic JIA | Whole Blood |
| GSM3056564 | Female | 4 | White | Systemic JIA | Whole Blood |
| GSM3056587 | Female | 6 | Black | Systemic JIA | Whole Blood |
| GSM3056589 | Female | 6 | Black | Systemic JIA | Whole Blood |
| GSM3056590 | Male | 13 | White | Systemic JIA | Whole Blood |
| GSM3056591 | Female | 9 | White | Systemic JIA | Whole Blood |

**Supplementary Table 1C. Characteristics of patients involved in qRT-PCR validation**

| **Sample_ID** | **Gender** | **Age (years)** | **Ancestry** | **Diagnosis** | **Tissue** | **Age at diagnosis (years)** | **Treatment** |
| --- | --- | --- | --- | --- | --- | --- | --- |
| HC1 | Male | 16 | Yellow | Control | Whole Blood | NA | NA |
| HC2 | Male | 5 | Yellow | Control | Whole Blood | NA | NA |
| HC3 | Male | 5 | Yellow | Control | Whole Blood | NA | NA |
| HC4 | Female | 11 | Yellow | Control | Whole Blood | NA | NA |
| HC5 | Male | 10 | Yellow | Control | Whole Blood | NA | NA |
| SJIA1 | Male | 16 | Yellow | Systemic JIA | Whole Blood | 10 | DMARDs, glucocorticoids, biologics, NSAIDs |
| SJIA2 | Male | 5 | Yellow | Systemic JIA | Whole Blood | 5 | NSAIDs |
| SJIA3 | Male | 5 | Yellow | Systemic JIA | Whole Blood | 4 | DMARDs, glucocorticoids, biologics, NSAIDs |
| SJIA4 | Female | 11 | Yellow | Systemic JIA | Whole Blood | 7 | DMARDs, glucocorticoids, biologics, NSAIDs |
| SJIA5 | Male | 10 | Yellow | Systemic JIA | Whole Blood | 10 | Glucocorticoids, NSAIDs |

DMARDs, disease-modifying antirheumatic drugs; NSAIDs, nonsteroidal anti-inflammatory drugs.
