# Supplementary figures and images for: Identification of Key Biomarkers and Immune Infiltration in Systemic Juvenile Idiopathic Arthritis by Integrated Bioinformatic Analysis
Source: Front Mol Biosci. 2021 Jul 14;8:681526. doi: 10.3389/fmolb.2021.681526 (PMC8316978; doi:10.3389/fmolb.2021.681526)

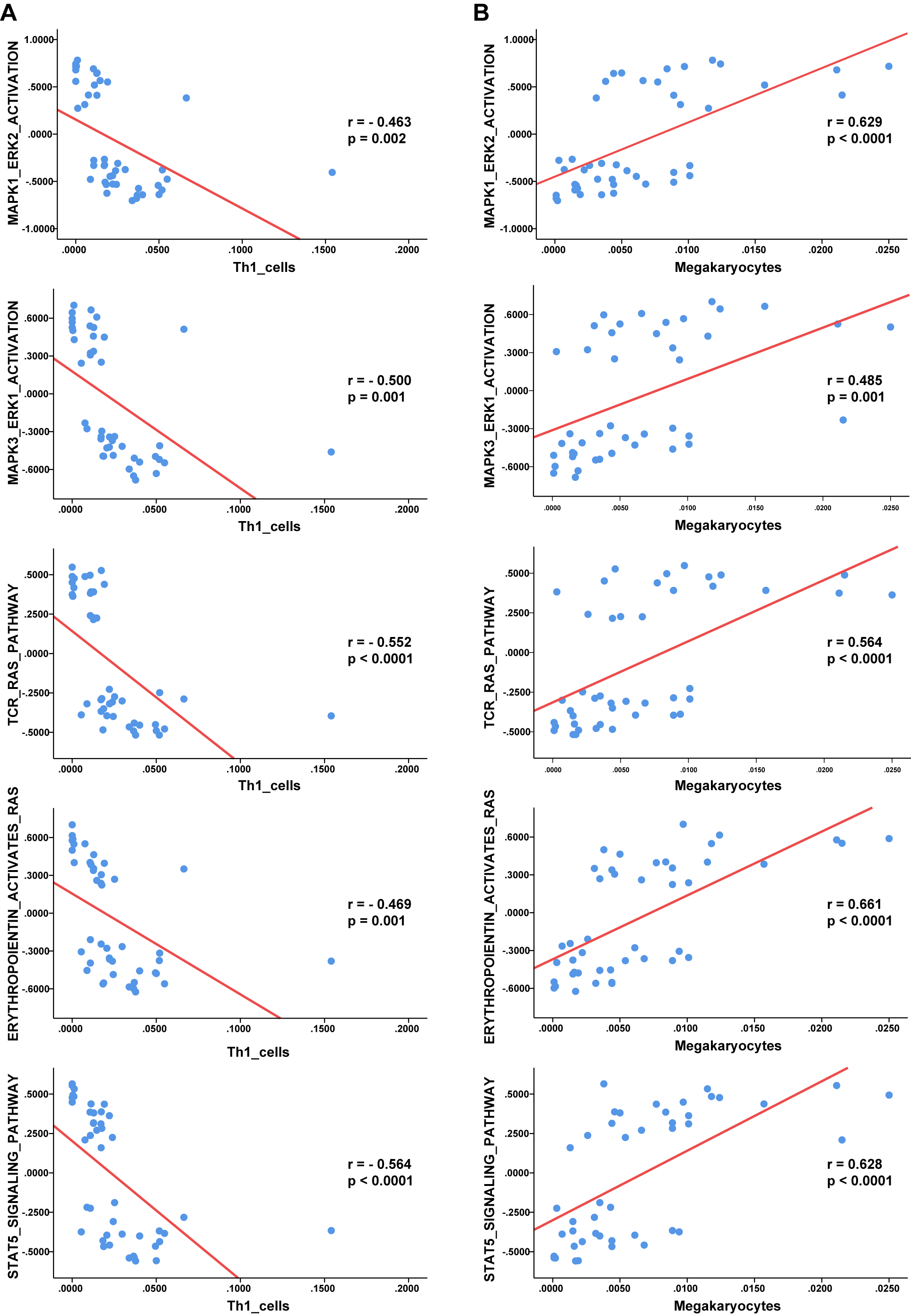

Supplement: Supplementary file 2 [file Image3.TIF]

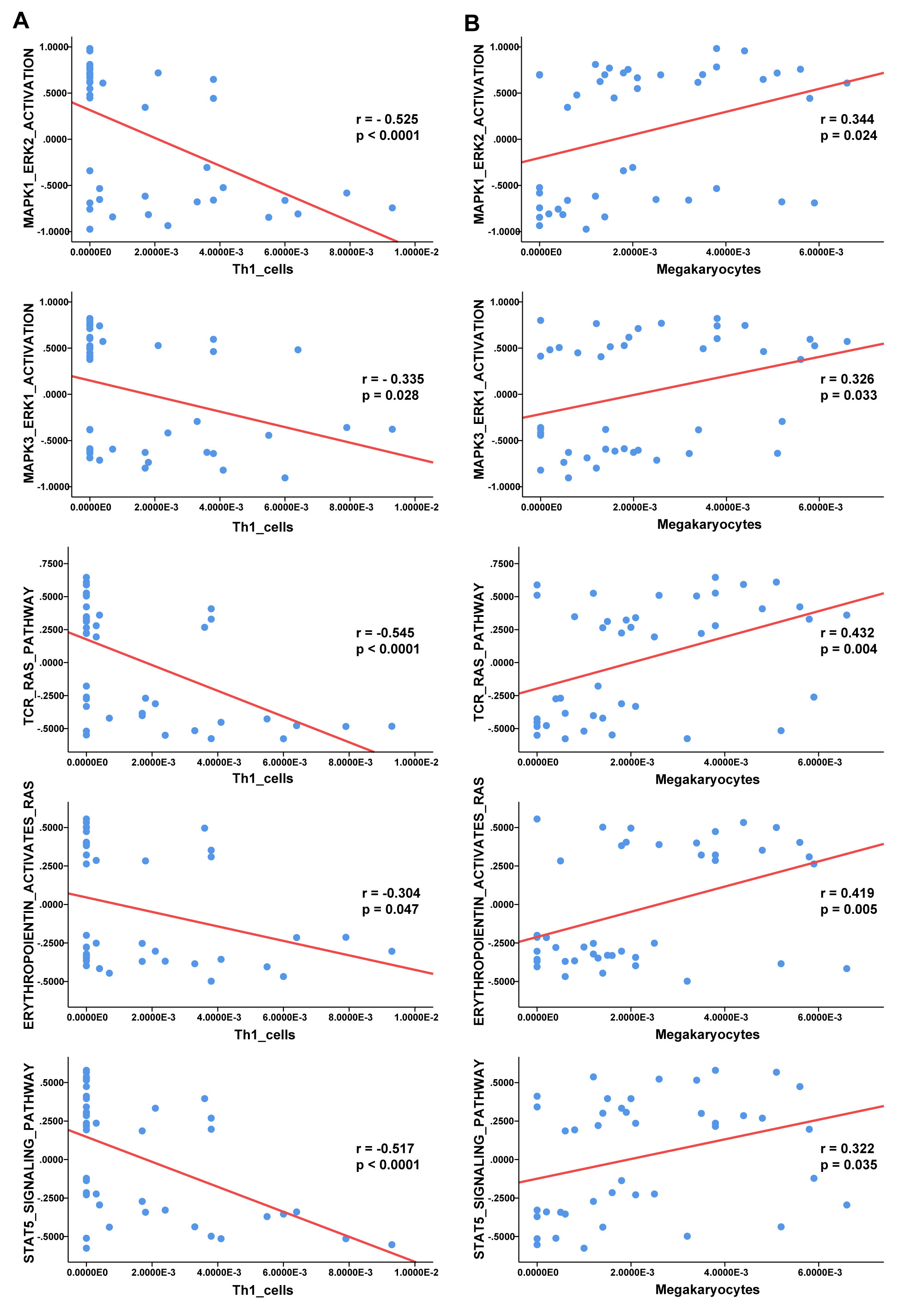

Supplement: Supplementary file 3 [file Image2.TIF]

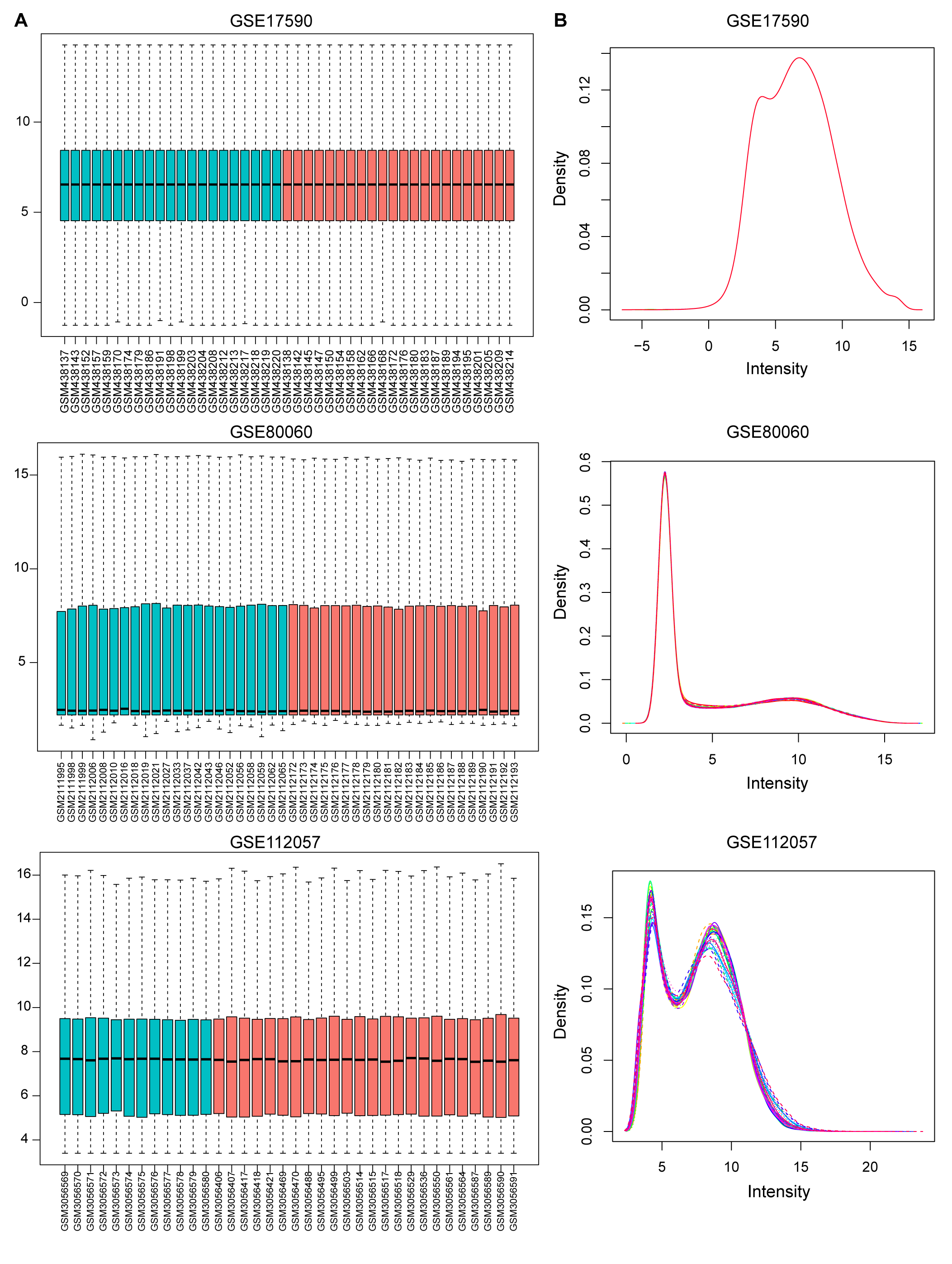

Supplement: Supplementary file 4 [file Image1.TIF]
