# Supplementary material for: Identification of Key Biomarkers and Immune Infiltration in Systemic Juvenile Idiopathic Arthritis by Integrated Bioinformatic Analysis
Source: Front Mol Biosci. 2021 Jul 14;8:681526. doi: 10.3389/fmolb.2021.681526 (PMC8316978; doi:10.3389/fmolb.2021.681526)
Supplement: Supplementary file 5 [file Table2.docx]

**Supplementary Table 2A. Primer sequence for qRT-PCR analysis**

| **Gene** | **Primers** | **Sequence (5’→3’)** |
| --- | --- | --- |
| ELANE | Forward primer | CGACCCCGTAAACTTGCTCAA |
|  | Reverse primer | CCCACGGTTCCTGCCCAGA |
| ARG1 | Forward primer | TGGACAGACTAGGAATTGGCA |
|  | Reverse primer | CCAGTCCGTCAACATCAAAACT |
| PGLYRP1 | Forward primer | GTGGGCTACAACTTCCTGATTG |
|  | Reverse primer | TGGGGTTCCATAAGTGACCTG |
| HPSE | Forward primer | TCATCAATGGGTCGCAGTTAGG |
|  | Reverse primer | TTAGCCGTCTTTCTTCGAGGC |
| CRISP3 | Forward primer | TACAGACACAGTAACCCAAAGGA |
|  | Reverse primer | TGGATTGCTTGTGACCATGAG |
| TCN1 | Forward primer | CAACTATGCGAGATTTGTGAGG |
|  | Reverse primer | CCAAAGCCAGTATAATCAAGGC |
| β-actin | Forward primer | GAGAAAATCTGGCACCACACC |
|  | Reverse primer | GGATAGCACAGCCTGGATAGCAA |

QRP-PCR, quantitative reverse transcription-polymerase chain reaction.

**Supplementary Table 2B. Procedure for qRT-PCR**

| **Temperature** | **Time** | **Cycle (s)** |
| --- | --- | --- |
| 95℃ | 2 minutes | 1 |
| 95℃ | 5 seconds | 40 |
| 58℃ or 60℃ | 30 seconds |  |
| Melt Curve 65℃ to 95℃ increment 0.5℃ | 5 seconds | 1 |
| END | | |

QRP-PCR, quantitative reverse transcription-polymerase chain reaction.
