# Supplementary material for: Identification of Key Biomarkers and Immune Infiltration in Systemic Juvenile Idiopathic Arthritis by Integrated Bioinformatic Analysis
Source: Front Mol Biosci. 2021 Jul 14;8:681526. doi: 10.3389/fmolb.2021.681526 (PMC8316978; doi:10.3389/fmolb.2021.681526)
Supplement: Supplementary file 6 [file DataSheet1.docx]

**Supplementary Figure Legends**

**Supplementary Figure 1 |** Quality assessment of the samples in each dataset. **(A)** Boxplots representing summaries of the signal intensity or read count distributions of the datasets. (**B)** Density plot showing density estimates of samples from each dataset.

**Supplementary Figure 2 |** Correlation analysis between immune cells and enriched pathways. (**A)** Analysis of the correlations of Th1 cells with the five enriched pathways in GSE17590. (**B)** Analysis of the correlations of megakaryocytes with the five enriched pathways in GSE17590.

**Supplementary Figure 3 |** Correlation analysis between immune cells and enriched pathways. (**A)** Analysis of the correlations of Th1 cells with the five enriched pathways in GSE80060. (**B)** Analysis of the correlations of megakaryocytes with the five enriched pathways in GSE80060.
